# Phenotypic flux: The role of physiology in explaining the conundrum of bacterial persistence amid phage attack

**DOI:** 10.1093/ve/veac086

**Published:** 2022-09-15

**Authors:** Claudia Igler

**Affiliations:** Department of Environmental Systems Science, ETH Zürich, Institute of Integrative Biology, Universitätstrasse 16, Zurich 8092, Switzerland

**Keywords:** phage-bacteria co-existence, phenotypic resistance, physiological heterogeneity, phenotypic flux, phage infection dynamics

## Abstract

Bacteriophages, the viruses of bacteria, have been studied for over a century. They were not only instrumental in laying the foundations of molecular biology, but they are also likely to play crucial roles in shaping our biosphere and may offer a solution to the control of drug-resistant bacterial infections. However, it remains challenging to predict the conditions for bacterial eradication by phage predation, sometimes even under well-defined laboratory conditions, and, most curiously, if the majority of surviving cells are genetically phage-susceptible. Here, I propose that even clonal phage and bacterial populations are generally in a state of continuous ‘phenotypic flux’, which is caused by transient and nongenetic variation in phage and bacterial physiology. Phenotypic flux can shape phage infection dynamics by reducing the force of infection to an extent that allows for coexistence between phages and susceptible bacteria. Understanding the mechanisms and impact of phenotypic flux may be key to providing a complete picture of phage–bacteria coexistence. I review the empirical evidence for phenotypic variation in phage and bacterial physiology together with the ways they have been modeled and discuss the potential implications of phenotypic flux for ecological and evolutionary dynamics between phages and bacteria, as well as for phage therapy.

## Introduction

Bacteria are omnipresent in our environment and our bodies, but they are still outnumbered by far by the viruses that prey on them, called bacteriophages. Accordingly, phages are likely to play a huge role in shaping our biosphere and in global biogeochemical processes; but they have also been crucial as key model systems in the fields of molecular biology and predator–prey coevolution (Campbell 1960; Levin, Stewart, and Lin 1977; Bremermann and Pickering 1983; Lenski and Levin 1985; Lenski 1988; Yoshida et al. 2007). In the face of rising antimicrobial resistance, interest in phage–bacteria dynamics has gained traction in recent years due to the possibility of using phages as antimicrobial therapies against bacterial pathogens (Levin and Bull 2004; McCallin and Brüssow 2017; Pires et al. 2020). Accordingly, we can build on more than 100 years of literature on empirical and theoretical studies investigating phage–bacteria ecology and evolution at the molecular and population levels, and yet, especially, efforts in predicting the success of phage therapy treatments have shown that there are still some puzzles remaining (Weld, Butts, and Heinemann 2004; Bull and Gill 2014).

One puzzle of particular interest is the persistence of largely sensitive bacterial cells in the face of ongoing lytic phage predation. Such coexistence has been observed *in vitro* (Chao, Levin, and Stewart 1977; Levin, Stewart, and Lin 1977; Lenski and Levin 1985; Lenski 1988; Fischer et al. 2004; Pisman and Pechurkin 2008) as well as *in vivo* (Smith and Huggins 1982; Hurley, Maurer, and Lee 2008; Weiss et al. 2009; Lourenço et al. 2020; Shkoporov et al. 2021). One of the main hypotheses proposed to explain this puzzle is the coevolution of bacterial resistance and phage counter-resistance (Weitz, Hartman, and Levin 2005; Koskella and Brockhurst 2014), even more so in light of the rampant discovery of new defense systems against mobile genetic elements (Egido et al. 2022). However, there are several other nongenetic explanations for how bacteria can find refuge from phage predation, which promote coexistence and strongly affect phage infection dynamics. It is crucial to understand such nongenetic refuges better in order to understand the selection pressures shaping phage and bacterial evolution. I will give a brief overview of different hypotheses explaining phage–bacteria coexistence before delving into the empirical evidence and mathematical descriptions of the most basic one: phenotypic variation in physiological traits of phages and bacteria.

## Mechanisms of phage–bacteria coexistence

While coexistence is less surprising or rather an in-built characteristic of temperate bacteriophages, which can integrate their own genome into that of the bacterial host cell, it is more difficult to understand coexistence with obligately lytic phages—which will be the focus of this review. To gather an intuition about potential mechanisms of coexistence, it is helpful to consider the interactions between phages and their bacterial hosts over a lytic phage cycle: phages infect susceptible host cells by adsorbing to a specific receptor on the cell surface, which is followed by injection of their genetic material. Subsequently, they hijack the biosynthesis machinery of the cell to replicate their DNA and produce viral proteins, which are then used to assemble phage virions and release them via cell lysis. Consequently, phage survival is dependent on:

- The presence of surface receptors allows for the recognition of host cells.
- The continued existence of a sufficient quantity of available hosts.
- The ‘quality’ of the host being high enough to support the synthesis and release of the phage particles.

There are five main hypotheses that can explain long-term phage–bacteria coexistence by striking a balance between phage infection efficacy being high enough to allow for phage survival but (s)low enough to also allow for bacterial survival. These hypotheses can be broadly divided into two categories based on the heritability of the coexistence mechanism.

### Genetic coevolution

One of the most prominent hypotheses is that featuring continuous phage–bacteria coevolution (Dennehy 2012; Koskella and Brockhurst 2014), which is supported by rapid resistance evolution against phages under laboratory conditions, for example, via mutations in phage receptors (Bohannan and Lenski 2000), and traces of frequent exchanges of phage tail fiber regions through horizontal gene transfer (Kutter et al. 1995). Coevolution is generally thought to be asymmetric, with phages evolving to adsorb to a different surface structure being more constrained than for bacteria to get rid of the phage receptor (Bohannan and Lenski 2000). However, this asymmetry will depend on the environment (e.g. the cost of losing the receptor), and continued coevolution has been observed with several phages and bacterial species (Koskella and Brockhurst 2014). It is also questionable if single mutations always give ‘complete’ resistance (Bohannan and Lenski 2000). Resistance can, for example, be partial at the single-cell level if complete resistance requires intermediate steps (Lenski 1984), or if receptors are not completely abolished (Spanakis and Horne 1987; Lythgoe and Chao 2003; Chapman-McQuiston and Wu 2008b; Høyland-Kroghsbo et al. 2013; Tan, Svenningsen, and Middelboe 2015), and at the population level through switching between resistant and susceptible phenotypes in genetically resistant bacteria (Chaudhry et al. 2018). In response to reduced receptor numbers, phages can decrease their adsorption specificity, which can however be costly due to increased rates of unsuccessful genome injection attempts (Schwartz 1980; Lenski and Levin 1985). Overall, continued coexistence via genetic coevolution alone seems unlikely because phage and bacterial evolution is generally limited by trade-offs (Bull 2006; Marianne and Taddei 2006; Wang 2006; Jover, Cortez, and Weitz 2013).

A special form of genetic coevolution includes the large diversity of bacterial defense systems and phage counter-defenses, which are currently being discovered (Hampton, Watson, and Fineran 2020). However, even in the absence of continuous coevolution, stable phage–bacteria coexistence can be possible due to regular loss of immunity with these systems (Weissman et al. 2018; Skanata and Kussell 2020).

### Nongenetic coexistence hypotheses

#### Numerical refuge

This explanation relies on the emergence of sufficient phage-resistant cells to dampen phage reproduction to an extent that allows the regrowth of susceptible cells, which can then maintain phages (Chao, Levin, and Stewart 1977; Levin, Stewart, and Lin 1977). In chemostat cultures, the coexistence of phages with susceptible and resistant bacteria was found to depend on a cost of resistance, i.e. slower growth of resistant cells, and on a continuous inflow of resources and outflow of phages (Chao, Levin, and Stewart 1977). The latter prevents unlimited phage growth, as phage decay is usually slow under laboratory conditions (Marianne and Taddei 2006). Phage decay can however be much higher under natural conditions, as was found in marine environments (Bratbak et al. 1996), for example, due to high UV exposure near the surface (Wommack et al. 1996). The coexistence of phages with resistant and susceptible cells was also observed in other *in vitro* (Kunisaki and Tanji 2010) and *in vivo* (Weiss et al. 2009) settings, with either resistant or susceptible bacterial subpopulations dominating.

#### Spatial refuge

Typically, phage–bacteria dynamics are studied in well-mixed environments, but many natural systems are more structured, offering bacteria a spatial refuge from phage predation (Schrag and Mittler 1996; Eriksen, Mitarai, and Sneppen 2020). Actively replicating bacteria in the gut microbiome are often associated with the mucus layer, for example (Chibani-Chennoufi et al. 2004; Lourenço et al. 2020), which shields them from phages. Even simple spherical growth of monoclonal bacterial colonies on agar plates can protect the cells growing on the inside from phage infection (Eriksen et al. 2017). Generally, many natural bacterial communities and pathogens form biofilms, which likely protect bacteria on the inside through decreased phage diffusion and other spatial effects that slow down phage infection (Briandet et al. 2008; Abedon 2017; Hansen et al. 2019). In fact, lower transmission rates in heterogenous environments, i.e. transient delays in phage spread, are sufficient to provide spatial refuge (Brockhurst, Buckling, and Rainey 2006; Abedon 2017; Simmons et al. 2018). Interestingly, the homogeneous control environments used by Brockhurst, Buckling, and Rainey (2006) also promoted coexistence in more than half of the replicates, but it was less stable than in heterogeneous environments. Even in well-mixed environments, adherence to the walls of culture tubes can enable phage–bacteria coexistence, although other unknown mechanisms might help in the stabilization of the effect (Schrag and Mittler 1996). In addition, in spatially structured environments bacterial motility can crucially affect coexistence, necessitating co-propagation of phages with moving bacteria (Li et al. 2020; Ping et al. 2020).

#### Phage inactivation

Bacteria can protect themselves from phage predation by transforming their environment in such a way that inactivates phage virions, for example, by producing ‘decoys’ for phage adsorption (Rabinovitch, Aviram, and Zaritsky 2003; Manning and Kuehn 2011) or proteolytic enzymes that damage phage virions (Tan, Svenningsen, and Middelboe 2015). The debris of lysed cells can ‘accidentally’ act as a decoy for phage adsorption, but some bacteria also actively produce outer membrane vesicles (OMVs) that contain receptors for phage adsorption. Decoys could potentially lead to the coexistence of phages and bacterial cells by acting as a sink for phages (Rabinovitch, Aviram, and Zaritsky 2003), but model simulations suggest that they have a minor effect (Bull et al. 2018). It is also unlikely that OMVs are produced solely for phage defense (Manning and Kuehn 2011). Decoys and proteolytic enzymes produce a similar effect in that they reduce the number of infective phage particles, but inactivation is likely only transient in the former case.

#### Physiological variation

Phenotypic variation in phage and bacterial physiology between and within environments can interfere with successful phage infection, for example, through changes in or loss of the bacterial cell wall (Ongenae et al. 2022). Physiological variation can be caused by intrinsic population heterogeneity, density-dependent changes, or other environmental influences.

*Population heterogeneity* can lead to variation in the number of surface receptors, which affects phage adsorption. Phenotypic switching between high and low numbers of receptors in a minority bacterial subpopulation was found to be capable of stably sustaining the survival of highly susceptible bacteria together with their phages (Chapman-McQuiston and Wu 2008a,b). While most coexistence mechanisms mainly consider adsorption, variation in physiological traits can interfere with the efficacy of all infection steps. After adsorption and DNA injection, phages redirect and take over a variety of intracellular processes (Jeroen et al. 2017; Jacobson, Callaghan, and Amador-Noguez 2021). All of these processes provide potential points of infection inhibition. *Bacterial density-dependent effects*, like metabolic shutdown during the stationary phase, can lead to insufficient support for phage particle synthesis (Tabib-Salazar et al. 2019). Interestingly, in contrast to most known phages, phage T7 replicates well in stationary phase cells (Schrader et al. 1997) due to its inhibition of the bacterial stationary phase RNA polymerase (Tabib-Salazar et al. 2018). In the absence of such phage-supplied mechanisms, however, starvation of bacterial hosts effectively inhibits their support of lytic phage development in well-mixed (Łos et al. 2007) and spatially structured environments (Li et al. 2020; Ping et al. 2020) alike. Population heterogeneity and density-dependent effects can be further influenced by *environmental conditions*, but I will only consider the direct effect of the environment on phage physiology via phage virion decay (Wommack et al. 1996; Marianne and Taddei 2006; Blazanin et al. 2022).

Box 1. A population model of phage–bacteria dynamics.

The complex eco-evolutionary interactions between phages and bacteria make mathematical models very useful tools in understanding and exploring their dynamics (Weitz 2016; Styles, Brown, and Sagona 2021). Most models used to describe phage infection dynamics are derivatives of the classical works (Campbell 1960; Levin, Stewart, and Lin 1977; Bohannan and Lenski 1997), which describe phage–bacteria ecology and coevolution in continuous culture (for summaries of different types of phage infection models see Stopar and Abedon 2008; Weitz 2016; García et al. 2019; Sinha, Grewal, and Roy 2020; Styles, Brown, and Sagona 2021). However, resource-limited environments, in which bacterial metabolism changes with the growth phase, are likely more relevant under most natural conditions. I will use a simple model of phage–bacteria dynamics in a well-mixed, resource-limited environment (based on Lenski and Levin 1985; Weitz 2016) to visualize the potential impact of variation in phage and bacterial physiology in the presence of genetic resistance evolution.

The model follows the number of susceptible *S*, infected *I,* and resistant *R* bacterial cells, as well as free phages *P*. Susceptible bacteria grow at a rate *r*, and resistant cells arise from *S* in a growth-dependent manner with mutation rate *µ* but carry a growth cost *c*. Infected bacteria *I* arise from *S* through phage adsorption. *I* cells do not grow, as lytic phage cycles are often relatively short and many phages inhibit host cell division. *S, I*, and *R* cells die (naturally) at the same rate *γ* and compete for a limited amount of resources, which allows for a cellular carrying capacity *K*. Free phages decay at rate *ω*, which is often much slower than the bacterial death (at least under laboratory conditions).

Assuming a well-mixed environment, phages adsorb to susceptible cells following mass–action kinetics at rate *α*. After a delay *τ*, which describes the latent period, during which phages replicate within the cell, infected cells are lysed and release a burst of *β* new phages. To account for the death of *I* cells over the latent period, the number of infections has to be modified by the probability *e*^− *γτ*^ to survive until burst (Herz et al. 1996). $\beta \alpha {e^{ - \gamma \tau }}S\left( {t - \tau } \right)P\left( {t - \tau } \right)$ then describes the release of *β* phages from cells that were infected *τ* time units ago and did not die in the meantime. Phages can adsorb to susceptible and infected cells, but secondary infection to *I* cells does not increase the phage burst and hence represents an additional phage death rate. Phages can however not infect resistant cells, as I am assuming that resistance stems from receptor loss. This leads to the following delay differential equations for phage–bacteria dynamics:

$$\frac{{dS}}{{dt}} = r\left( {1 - \mu } \right)S\left( {1 - \frac{{S + I + R}}{K}} \right) - \alpha SP - \gamma S$$

$$\frac{{dI}}{{dt}} = \alpha SP - \alpha {e^{ - \gamma \tau }}S\left( {t - \tau } \right)P\left( {t - \tau } \right) - \gamma I$$

$$\frac{{dR}}{{dt}} = r\left( {1 - c} \right)R\left( {1 - \frac{{S + I + R}}{K}} \right) + \mu rS\left( {1 - \frac{{S + I + R}}{K}} \right) - \gamma R$$

$$\frac{{dP}}{{dt}} = \beta \alpha {e^{ - \gamma \tau }}S\left( {t - \tau } \right)P\left( {t - \tau } \right) - \alpha \left( {S + I} \right)P - \omega P$$

Using this model to deterministically simulate a phage epidemic with empirically realistic parameters for well-mixed laboratory conditions (Table S1), I find that phages and susceptible cells will die out (i.e. drop below one cell or phage), while resistant bacteria rise to carrying capacity if they emerge fast enough (Figs 2A and S1A). Note that in other parameter regimes (e.g. high resistance cost and fast phage decay) this model can also predict the coexistence between phages and bacteria (Weitz 2016), but I will use the parameter set in Table S1 to study the potential for the emergence of coexistence. Furthermore, the dependence of predicted dynamics on the relative magnitude of phage and bacterial parameter traits (Weitz 2016) already suggests the importance of phenotypic variation in these traits for coexistence.

These five explanations for phage–bacteria coexistence are not mutually exclusive. In particular, spatial and physiological effects can be directly linked, for example, when considering phage predation of biofilms (Abedon 2017; Hansen et al. 2019) or micro-colonies (Eriksen et al. 2017), where slowdown of phage diffusion together with population heterogeneity can obviate the need for genetic resistance (Eriksen, Mitarai, and Sneppen 2020; Attrill et al. 2021). However, given their different underlying mechanisms and resulting dynamics, it is useful to first study each refuge individually. Here, I focus on phenotypic variation in phage and bacterial physiology, particularly within one environment, which might play a bigger role in phage–bacteria coexistence than currently appreciated.

## Phenotypic flux through physiological variation

Nongenetic, physiological variation in phage and bacterial traits has been empirically observed even in the simplest of set-ups, i.e. well-mixed cultures started from a single isolate. This indicates that most (clonal) phage and bacterial populations will be in continuous phenotypic flux.

**Figure 1.**
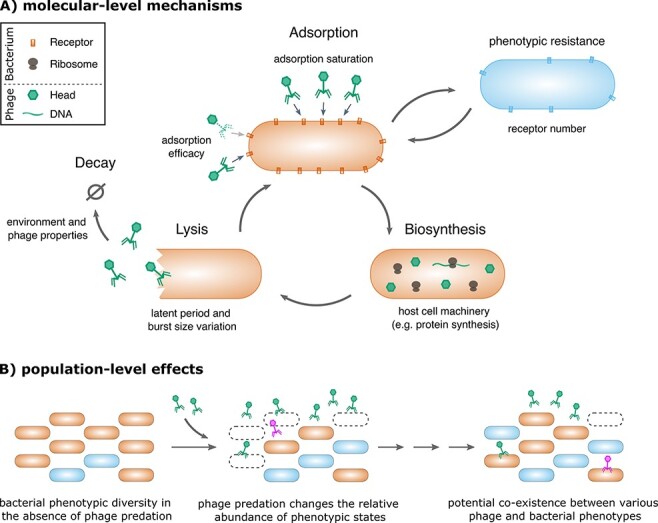
Phenotypic flux in phage infection dynamics. (A) Molecular mechanisms producing phenotypic flux: phage adsorption depends on the number of receptors at the bacterial cell surface, which varies within and between environments, potentially leading to phenotypic resistance. Adsorption can be lower in phage subpopulations with reduced adsorption efficacy (dashed tail fibers) and will be ‘effectively’ reduced by saturation as more phages adsorb to one cell. Phage replication and protein production (biosynthesis) within the cell rely largely on the bacterial machinery, potentially leading to variation in phage numbers produced (burst size) or time to lysis (latent period). Phage decay outside of the cell strongly depends on phage properties and the environment. (B) Phenotypic flux, i.e. a distribution of phenotypic states connected by nongenetic changes, exists in the bacterial population in the absence of phage predation (for simplicity only two states are shown but represent a potentially large variety of states). Phage predation changes the relative abundance of bacterial phenotypic states according to differences in susceptibility and can additionally lead to a variety of phage phenotypic states. Long-term, phenotypic flux can reduce the force of phage infection and allow for stable coexistence of various phage and bacterial states.

‘Phenotypic flux’ is the distribution of different physiological states in a phage or bacterial population, and each state is connected to other states through nongenetic changes, for example, through changes in gene expression or protein number (Fig. 1). Flux between states can occur during the life cycle or at ‘birth’ of a phage or bacteria. A distribution of bacterial states exists also in the absence of phages, but the presence of phages will change the distribution in relation to the susceptibility of bacterial states to phage infection. This new distribution of physiological states can be largely self-sustaining in the presence of phages and can result in stable coexistence.

The existing knowledge about mechanisms producing a variation in physiological states relevant for phage infection is however currently largely disconnected from describing the resulting infection dynamics at the population level. Experimental designs of phage infection experiments are often not suitable for identifying phenotypic variation, which is challenging due to its transient nature (Bull et al. 2014). However, understanding how phenotypic flux affects the interplay between phage growth and bacterial killing could be a key to understanding phage–bacteria ecology and evolution (Choua et al. 2020). I will demonstrate this idea by connecting empirical evidence of phenotypic variation in phage and bacterial physiologies with their potential impact on population dynamics by using a simple mathematical model (Box 1).

### Adsorption: population heterogeneity

Phage adsorption to the bacterial cell envelope is the first step in phage infection and a crucial determinant of infection dynamics (Rodin and Ratner 1983; Hurley, Maurer, and Lee 2008; Shao and Wang 2008). Generally, what is thought of as adsorption rate is however more of an ‘infection’ rate and subsumes several biological processes: extracellular phage diffusion, encounter and attachment of a cell receptor, and genome translocation into the host cell cytoplasm (Dennehy and Abedon 2021). Accordingly, adsorption efficacy will likely be affected in various ways by changes in phage or host cell physiology, and it is, for example, unclear if the rate increases (Hadas et al. 1997; Schenk and Sieber 2019), decreases (Nabergoj, Modic, and Podgornik 2018), or remains constant (Golec et al. 2014) with faster bacterial growth. I will mainly discuss the influence of physiological variation on adsorption through the attachment of phages to cell receptors (Fig. 1), which has been studied thoroughly and empirically.

Phage attachment to the host cell can be extremely efficient at the population level, as typical phage adsorption rates measured in laboratory environments are close to the theoretical limit defined by diffusion (Schwartz 1976; Berg and Purcell 1977). However, at the individual level, ‘adsorption efficacy’ was found to differ within a phage population (Storms et al. 2010, 2012; Storms and Sauvageau 2015). Particularly, a subpopulation of phages can show reduced receptor affinity (Delbruck 1940), with the size of this subpopulation differing between phage species and environments (Storms et al. 2010, 2012; Storms and Sauvageau 2015). Reduced receptor affinity can be beneficial in environments, where adsorption is detrimental (Gallet, Lenormand, and Wang 2012). This is exemplified by phage T4, which can retract the tail fibers needed for adsorption (Golec et al. 2011) under conditions that are potentially unfavorable for reproduction (Conley and Wood 1975). A low-adsorbing phage fraction can be described mathematically by using two phage populations with high and low adsorption rates, respectively. This description assumes that the same fraction is produced at each burst, e.g. 20 per cent of the phages in a burst do not adsorb efficiently (Supplementary Equations (1–5)). The effect is similar to reducing the burst size, slightly delaying phage infection dynamics initially, but the low-adsorbing fraction would only become relevant when most of the phages have already adsorbed to host cells (Fig. S1A). The adsorption rate could also be described as a distribution function or by making it time-dependent to account for a gradual decrease in adsorption efficacy over the lifetime of a phage virion, but these effects are likely to be similarly small under the conditions considered here.

Bacterial physiology affects phage adsorption efficacy through potentially substantial variation in the ‘number of receptors’ presented on the surface between cells within a population (Chapman-McQuiston and Wu 2008a). Such variation can be caused by environmental conditions as many phage receptors are transmembrane proteins that are differentially expressed in the presence of certain biomolecules and because receptor number scales with bacterial cell size (Schenk and Sieber 2019). Even within a given environment, however, population heterogeneity can lead to differences in the production and accessibility of receptors, protecting a subpopulation of cells against phages. This effect is called ‘transient (phenotypic) resistance’ (Bull et al. 2014) and can be intrinsic, stemming from noise in gene expression (Chapman-McQuiston and Wu 2008a), or potentially adaptive, caused by phase variation (Kim and Ryu 2012; Turkington et al. 2019), quorum sensing (Høyland-Kroghsbo et al. 2013; Tan, Svenningsen, and Middelboe 2015), stress responses (Tzipilevich et al. 2022), plasmid- or phage-mediated modifications (Riede and Eschbach 1986; Decker et al. 1994; Bondy-Denomy et al. 2016), or genomic instability (Chaudhry et al. 2020). The relatively fast reversal of phenotypic resistance—as compared to genetic alterations—can alleviate its cost and is particularly favorable in changing environments (Brockhurst, Buckling, and Rainey 2005). Phenotypic resistance can be incorporated into mathematical models by considering the variation in receptor number. This could be done either implicitly, e.g. in correlation with cell surface area changes (Supplementary Equation (6); Schenk and Sieber 2019), or explicitly, by introducing bacterial subpopulations with different receptor numbers (Supplementary Equation (7); Chapman-McQuiston and Wu 2008b).

**Figure 2.**
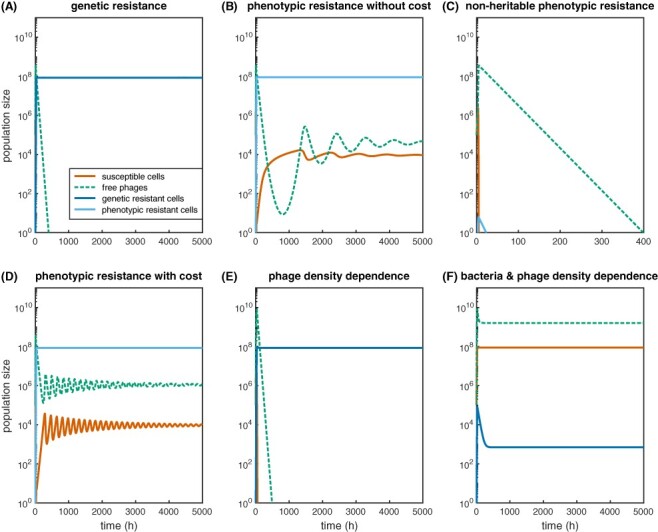
Phage–bacteria population dynamics considering phenotypic flux in phage adsorption. Phage and bacterial numbers (PFU/ml and CFU/ml) were simulated for 5,000 h starting from an initial MOI = 1 (other parameters are given in Table S1). Population dynamics are shown for constant adsorption rate with (A) genetic (stable) resistance; (B and C) phenotypic resistance without cost and switching of bacterial cells between high- and low-adsorbable states at (B) intermediate or (C) fast rates; and (D) phenotypic resistance with intermediate switching and growth cost. (E) and (F) show genetic resistance evolution when the adsorption rate is (E) phage- or (F) phage- and bacterial density-dependent. Orange indicates susceptible cells, dashed green indicates free phages, dark blue indicates genetically resistant cells in A, E, and F; and light blue indicates phenotypically resistant cells in B–D.

More generally, by disregarding mechanistic aspects and focusing on the dynamics, phenotypic resistance can be described mathematically using one or more transiently low-adsorbable bacterial subpopulation(s). As compared to genetic resistance, phenotypic resistance will lead to back-and-forth switching between the *R* and *S* states at rates *s_f_* and *s_b_* (Supplementary Equations (8–11)) that are generally faster than mutation rates. These switching rates govern the stability of phenotypic resistance and are likely dependent on the underlying mechanism (Bull et al. 2014). Considering, for example, a lower number of receptors, the *R* cells will be partially resistant, allowing phage adsorption at a lower rate *α_l_ < α* but will otherwise support the same latent period and burst size as infected *S* cells. If phenotypic resistance is a ‘by-product’ of cellular heterogeneity and does not induce a growth cost *c*, phenotypic resistance can lead to long-term stable coexistence between both bacterial populations (*S* and *R*) and phages (Figs 2B and S2B). This agrees with *in vitro* and *in vivo* observations of phages and bacteria that display phase variation in cell surface structures (Shkoporov et al. 2021). My model suggests that stable coexistence is dependent on the replenishment of susceptible bacteria through back-switching from resistance (which is similar to the empirical results in Chaudhry et al. 2018). Without back-switching, the resistant cells take over, while phages and sensitives go extinct—regardless of whether resistance is complete or partial (Figs 2A and S1B). If, on the other hand, back-switching is very fast, e.g. phenotypic resistance is lost at every cell division, then the phenotypic subpopulation cannot protect the entire population, and bacteria, as well as phages, go extinct (Figs 2C and S2C). Hence, in this parameter regime, intermediate phenotypic switching rates produce stable phage–bacteria coexistence, which is facilitated by, but not dependent on, the fact that phenotypic resistance is partial (Fig. S1C).

In particular, if phenotypic resistance is an adaptive mechanism against phage predation, it might also confer a growth cost. Including a cost of phenotypic resistance allows for more regrowth of *S* cells after the first wave of phage infection, which then causes faster oscillations in the phage and susceptible populations, which diminish over time to give stable coexistence (Figs 2D and S2D). These oscillations are caused by the feedback between phage and host cell growth. Notably, the frequency of the oscillations is determined by the magnitude of the resistance cost, with faster oscillations for higher costs.

### Adsorption: density-dependent variation

The majority of phages known are affected by the bacterial growth phase (Hadas et al. 1997). The number of free phages establishing a successful infection generally decreases as metabolism slows down when bacteria approach the stationary phase. This is most often incorporated into mathematical models by modifying the adsorption rate (Supplementary Equations (12–15); Schrag and Mittler 1996; Weitz and Dushoff 2008; Wang and Goldenfeld 2010; Santos et al. 2014; Krysiak-Baltyn, Martin, and Gras 2018), even though receptor attachment might not be the (only) infection step inhibited in stationary phase (Storms and Sauvageau 2015; Qin et al. 2017). However, bacterial cells tend to shrink during the stationary phase (Akerlund, Nordstrom, and Bernander 1995), making them smaller targets with presumably less receptors. In the base model (Box 1), this effect would only play a role if susceptible bacteria could survive long enough to enter the stationary phase (Fig. S1D). Therefore, making the adsorption rate ‘bacterial density-dependent’ (Supplementary Equation (13)) by itself does not change the infection dynamics.

The effective adsorption rate of the phage population will however decrease not only with bacterial but also with phage density. This stems from a ‘lack of physiological change’, meaning that the number of phages produced by one host cell does not increase if more than one phage infects the cell. Therefore, at the population level, the rate of ‘successful’, i.e. burst-producing, infections will saturate as the phage-to-bacteria ratio, the multiplicity of infection (MOI), becomes much larger than 1. (Notably, coinfections of the same phage can lead to complex intracellular interactions, affecting the burst size and latent period (Ellis and Delbrück 1939; Doermann 1948; Gadagkar and Gopinathan 1980; Abedon 2019), which likely influences infection dynamics but is little explored so far.) ‘Saturation of phage (adsorption) efficiency’ can be described mathematically by using, e.g. a saturating Hill function to make the adsorption rate phage density-dependent (Supplementary Equations (16 and 17); Smith 2008; Stopar and Abedon 2008; Rodriguez-Gonzalez et al. 2020). This effect results in stable coexistence with a high number of phages and susceptible cells in the absence of genetic resistance evolution (Fig. S1E), but the dominance of the resistant strain and extinction of phages otherwise (Figs 2E and S2E). Coupled phage- and bacterial density-dependence of adsorption, however, leads to stable coexistence with high susceptible and phage numbers in the presence and absence of resistance (Figs 2F, S1F, and S2F).

Note that while I am only concerned with well-mixed environments here, it is possible to modify adsorption to introduce spatial heterogeneity implicitly into the model (Rodriguez-Gonzalez et al. 2020) by using a power-law exponent (Supplementary Equation (18)). (For a more detailed discussion on modeling spatial structure in phage infections, see Bull et al. 2018.)

Overall, empirical data demonstrates a complex functional dependence of adsorption efficacy on phage and bacterial physiologies and densities, which can substantially alter model predictions of phage–bacteria dynamics. The probability for stable coexistence is increased for transient, partial resistance, and ‘self-regulation’ of the system via saturation of phage infection efficacy at high phage-to-bacteria ratios.

### Biosynthesis: density-dependent variation

After phage genome injection into the host cell, the host cell machinery is taken over and redirected to synthesize new phage virions (Fig. 1; Krueger and Schroeder 1981; Roucourt and Lavigne 2009; Van Den Bossche et al. 2014). The importance of the resulting dependence on host cell metabolism—specifically on replication, gene expression, and protein synthesis capability (Hadas et al. 1997; You, Suthers, and Yin 2002; Qin et al. 2017; Hsueh et al. 2022)—for phage fitness is highlighted by the finding that some phages carry their own transcription systems (Chamberlin, Mcgrath, and Waskell 1970; Maitra 1971; Ceyssens et al. 2014), even though this might be costly due to a larger genome. Most known phages are unable to productively infect metabolically inactive bacteria (Meeske, Nakandakari-Higa, and Marraffini 2019), but more generally, decreased growth rates lead to reduced burst sizes and longer latent periods (Kokjohn and Sayler 1991; Hadas et al. 1997; Schrader et al. 1997; Middelboe 2000; You, Suthers, and Yin 2002; Golec et al. 2014; Nabergoj, Modic, and Podgornik 2018). For phage T4, for example, burst size can change over an order of magnitude and latent period, which is the time spent within the bacterial cell, can become four times longer (Nabergoj, Modic, and Podgornik 2018). Both traits show a non-linear and saturating correlation with growth rate (Supplementary Equations (19–24); Nabergoj, Modic, and Podgornik 2018; Choua and Bonachela 2019).

**Figure 3.**
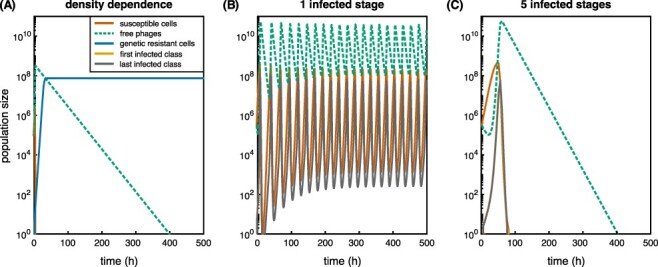
Phage–bacteria dynamics for bacterial density-dependent burst size and realistic latent period distributions. Phage and bacterial numbers (PFU/ml and CFU/ml) were simulated for 500 h starting from an initial MOI = 1 (parameters are given in Table S1). Population dynamics are shown for (A) bacterial density-dependent burst size with resistance evolution; (B and C) gamma-distributed latent periods with (B) one or (C) five infected stages without resistance evolution (see Fig. S3 for (B) and (C) with resistance evolution). For (B) and (C), lower adsorption rate (*α* =10^−9^) and higher phage decay rate (*ω* =0.33) were used. Orange indicates susceptible cells, dashed green indicates free phages, and dark blue indicates genetically resistant cells. In (B) and (C), yellow shows the first infected stage and gray shows the last one (both overlap with the orange S curve).

I find that ‘bacterial density-dependence’ of burst size (using the same functional form of bacterial density-dependence as for adsorption, Supplementary Equation (13)) does not change infection dynamics substantially (Fig. 3A). Such a dependence is likely to only play a significant role if it slows down the phage epidemic enough for bacteria to reach stationary phase and enter a less metabolically active state. More complex models incorporating metabolic flux balance analysis can however be useful to quantitatively investigate the limiting steps for phage production within the cell (Birch, Ruggero, and Covert 2012).

### Latent period: population heterogeneity

The latent period determines the time that a phage spends on linear reproduction within a cell, i.e. the burst size (Kannoly et al. 2021), as opposed to the potential geometric increase attained by infecting new cells. Therefore, latent period is an important fitness determinant that should be optimized in a given environment (Wang 2006). The surprising failure to evolve phages toward the predicted optimal latent period and burst size (Heineman and Bull 2007; Chantranupong and Heineman 2012) could indicate that while these traits are correlated, they might depend in different ways on host physiology. For example, bacterial growth rate variations affect both traits but not to the same extent (You, Suthers, and Yin 2002; Golec et al. 2014; Nabergoj, Modic, and Podgornik 2018; Schenk and Sieber 2019). Furthermore, latent periods vary little between infected cells within a given environment (Cahill and Young 2019), while burst sizes follow much wider distributions (Delbruck 1945; Dennehy and Wang 2011; Santos et al. 2014), suggesting that wider latent period distributions might be disadvantageous.

I explored the effect of ‘latent period distributions’ by describing the infection delay in the mathematical model (Box 1) with one or more stage(s) of phage-infected bacteria (instead of using *τ*). I assume that only the last infected stage produces a phage burst (Supplementary Equations (25–31); Lloyd 2001b). By adjusting the transition rates between the stages according to the number of stages used, the mean latent period is kept the same, but the distribution becomes narrower with the increasing number of infected stages. This approach results in a gamma distribution, but especially for a large number of infected stages, a normal distribution could be used as well (Rabinovitch et al. 2002; Santos et al. 2014). To investigate the influence of latent period distribution on coexistence, I am using faster phage decay and lower adsorption rates, which allows for the coexistence of phages and susceptible bacteria with one infected stage in the absence of resistance. Narrower distributions at higher numbers of stages lead to larger amplitudes in the oscillations of phage and bacterial populations. This can change the dynamics from coexistence between susceptible bacteria and phages to extinction of both (Fig. 3B, C). Allowing for (genetic) resistance to evolve, phages always go extinct and susceptible bacteria only survive for wide latent period distributions (Fig. S3A, B). Although the specific dynamics will depend on the relative parameters of the system (for more detailed analyses of eco-evolutionary consequences of latent period changes, see Bonachela and Levin 2014; Weitz 2016), overall, narrower distributions increase population oscillations and the probability of extinction, which suggests that stochastic effects will become more important.

Here, I assumed that all cells produce the same number of phages at lysis, but the model can be extended to include different burst sizes depending on the time of lysis, i.e. smaller burst sizes for lysis in earlier infected stages. In this case, very short latent periods would be disadvantageous as they would lead to few or even no phage progeny released, which could explain the low variance observed in empirical measurements of latent period distributions (Cahill and Young 2019). Except for very short latent periods, however, the variation in burst size is potentially only weakly influenced by variation in latent period (Kannoly et al. 2021).

Even though relatively few modeling studies consider variations in latent period and burst size (Supplementary Equations (19–34); Schrag and Mittler 1996; Smith and Thieme 2012; Santos et al. 2014; Krysiak-Baltyn, Martin, and Gras 2018; Nabergoj, Modic, and Podgornik 2018; Choua and Bonachela 2019; Schenk and Sieber 2019), phage infection dynamics are sensitive to these variations (Kerr et al. 2008; Smith and Thieme 2012; Weitz 2016; Figs 3B, C and S3), and capturing the natural distributions in these traits might be key to reproducing empirical observations of coexistence.

### Decay: phage and environmental variation

After being released from the host cell via lysis, free phage virions need to remain viable until they find the next host, which is determined by their decay rate. Virion decay is largely ignored under laboratory conditions, as they are assumed to be highly stable. However, this parameter might be higher than expected (Abedon 2009b) and varies substantially between different phages in correlation with their ‘physical properties’ like packaged DNA density (Marianne and Taddei 2006). In addition, decay rates are likely to be much more variable in ‘natural environments’. For example, they can be increased due to UV light exposure (Bratbak et al. 1996; Wommack et al. 1996), inactivation by the immune system (Dabrowska and Abedon 2019), or other stressors within the human body (Blazanin et al. 2022). Within a constant environment, the average phage decay is exponential, i.e. the probability of inactivation does not increase over time (Marianne and Taddei 2006), but it is unclear if and how much phage virion decay varies within a phage population. Hence, depending on the environment and phage movement through it, phage decay rates could be described mathematically as constants or as more complex distributions. Generally, faster decay rates decrease the force of phage infection and increase the probability of bacterial survival. This can shift coexistence due to phenotypic resistance in phage adsorption from intermediate to high switching rates (Figs S4B–D vs. 2B–D). It can further allow for coexistence when adsorption rate saturates at high phage densities, even in the presence of genetic resistance (Figs S4E vs. 2E), highlighting the importance of relative phage and bacterial population turnover (Weitz 2016) and the necessity to reflect realistic time scales in mathematical descriptions.

## Phenotypic flux shapes phage–bacteria ecology and evolution

While phages and bacteria can coevolve for many generations (Weitz, Hartman, and Levin 2005; Dennehy 2012; Koskella and Brockhurst 2014), the whole picture of coexistence is likely more nuanced: phenotypic flux due to physiological variation in phage and bacterial populations can dampen the force of infection and thereby lower the selection for resistance evolution—potentially to an extent where the costs outweigh the benefits (Fig. 2F). Hence, in order to understand phage evolution, it is paramount to first understand phage ecology.

Similar to the numerical refuge hypothesis, coexistence between phages and bacteria is possible if physiological variation limits phage infections sufficiently to allow the continued survival of susceptible bacteria. Coexistence due to physiological variation is facilitated by:

- Transient (genetic or nongenetic) resistance with intermediate switching rates between susceptible and resistant states.
- Growth costs of phenotypic resistance.
- Saturation of phage infection efficiency at high phage densities.
- Bacterial density-dependence of phage infection efficiency (combined with slowdown of phage infection by another mechanism).
- Higher variance of latent period distributions.
- Intermediate phage turnover rates.

The physiological nature of phage inhibition discussed here leads to very different dynamics and selection pressures than stable—and hence likely genetic—bacterial resistance to phages. In particular, for mathematical descriptions, resistance to phages is typically assumed to be stable and complete, while resistance can come in several flavors, from genetic to phenotypic and complete to partial (Bohannan and Lenski 2000). Although partial or transient resistance is not obviously favorable over stable resistance due to the remaining killing, it might still be more likely to occur if it is less costly or easier to obtain. Genetic resistance can be quite costly as it often involves the loss of receptors needed for the uptake of metabolites. Phenotypic switching is generally faster than mutation rates, making it potentially the less costly solution due to the fast restoration of function (Chaudhry et al. 2018; Turkington et al. 2019; Skanata and Kussell 2020). Furthermore, bacteria are likely to encounter more than one phage under natural conditions, which can make phenotypic resistance the most attainable solution, as for example shutdown of cellular metabolism will protect against most known phages. Depending on the specific mechanism, phenotypic resistance might be less of an adaptive mechanism against phage predation per se than a ‘by-product’ of a function that has evolved for a different reason but increases survival in the presence of phages as well (e.g. phase variation in cell surface structures).

While phenotypic resistance slows down phage infection, it might not drive phages extinct, thereby reducing the selection pressure on phages to evolve higher virulence as compared to genetic resistance. The type of resistance will determine the evolutionary potential for phage escape and counter-evolution (Hofnung, Jezierska, and Braun-Breton 1976). The effect of phenotypic flux on infection dynamics leads to the selection of traits that are not directly involved in the ‘typical’ phage–bacteria coevolution, namely physiological traits. Phage predation could, for example, select slow-growing subpopulations with lower metabolic activity and decreased cell dimensions, slowing down phage biosynthesis and adsorption, respectively (Schenk and Sieber 2019; Ping et al. 2020). For phages, on the other hand, it could be beneficial to carry genes that alleviate their strong dependence on host cell metabolism, for example, by bringing their own synthesis machinery (Chamberlin, Mcgrath, and Waskell 1970; Maitra 1971; Ceyssens et al. 2014; Tabib-Salazar et al. 2018). Other strategies to deal with non- or slow-growing host populations are phage dormancy modes like lysogeny and pseudolysogeny (Abedon 2009a; Maslov and Sneppen 2015), recycling of host components for phage reproduction (Bryan et al. 2016), increased environmental phage virion stability (Marianne and Taddei 2006), and avoidance of stationary phase infection (Golec et al. 2011). Hence, while only obligately lytic phages are considered here, the described concepts apply to temperate phages to a certain extent as well or rather help to explain why a temperate lifestyle is beneficial. Lysogeny constitutes an ‘extreme form’ of plasticity, i.e. physiological variation, that can be found in phage latent period between environments (Dennehy and Wang 2011). Hence, physiological variation in phage traits could be more beneficial in response to bacterial phenotypic flux than evolving an optimal trait according to the average host phenotype. The relative fitness costs and benefits of various phage strategies to deal with phenotypically suboptimal host states will depend on the duration and frequency of encountering these states and deserve a closer investigation in future work on phage infection dynamics.

## What does phenotypic flux mean for phage therapy?

In pathogen infections, bacterial cell states are likely to be highly variable and in constant phenotypic flux, given their need to evade the immune system and to adapt to the often stressful conditions encountered at an infection site (Tsai and Coombes 2019). Such phenotypic flux substantially increases the difficulty of predicting the success of phages as antimicrobial treatments. Extrapolation from phage characterization under laboratory conditions to the human environment is likely not straightforward as the impact of variation in some physiological traits is highly dependent on changes in other traits (Fig. 2E, F). Does that mean phage therapy is doomed? I would argue it is not, but that we have to extend our current approaches to it in the following manner:

- The role of physiological variation present in bacterial pathogen populations requires more consideration, particularly under clinically relevant conditions, as it can affect phage-killing efficacy in the presence and absence of genetic resistance (Figs 2 and S2).
- All infection steps are important for killing of the bacterial cell, therefore the focus should be extended more beyond studying adsorption (Egido et al. 2022). Phage therapy cocktails could, for example, be designed in a manner that broadens the range of targeted bacteria in terms of attachment sites, but also in terms of dealing with different metabolic states of host cells (e.g. replication on stationary phase bacteria (Tabib-Salazar et al. 2018; Maffei et al. 2022)).
- Phage decay is the only phage trait independent of the bacterial state, but it can affect infection dynamics substantially (Fig. S4). Hence, synthetic engineering efforts to optimize phage stability in clinical settings (Merril et al. 1996; Favor et al. 2020) constitute a promising avenue for improving phage therapy efficacy.
- Mathematical models are crucial tools in advancing our design of phage therapies (Payne and Jansen 2001; Cairns et al. 2009; Delattre et al. 2022) by allowing us to extend molecular knowledge to population dynamics predictions. However, currently used models often oversimplify phage infection processes, which limits their relevance in clinical settings. A more realistic model of phage–bacteria dynamics (Box 2) might be necessary to predict phage therapy success (see Table 1 and next section for empirical data needed to identify appropriate model structures).

**Table 1.** Impact and empirical evidence of physiological variation in phage infection processes.

Infection process	Physiological mechanism	Effect on coexistence	Empirical data available	Empirical data missing
Adsorption	Phage subpopulation with lower adsorption efficacy	Likely minor as this subpopulation only plays a role once the efficient phages have adsorbed	Proportion of different phage populations failing to adsorb for different media conditions (Storms et al. 2010, Storms et al. 2012); intrinsic variation in (average) adsorption between different phages (Marianne and Taddei 2006)	Dynamics of low-adsorbing phage subpopulations over time and ‘heritability’ of the subpopulation size
Adsorption	Phenotypic resistance due to population heterogeneity (noise) in the number of receptor molecules on the bacterial cell surface	Increases the probability of coexistence between phages and bacteria of low- and high-susceptibility	Phage Lambda receptor number heterogeneity within an *E. coli* population for different media conditions (Chapman-McQuiston and Wu 2008a)	Transition rates between subpopulations with different receptor numbers
	Phenotypic resistance due to other mechanisms of switching between subpopulations	Increases the probability of coexistence between phages and bacteria of low- and high-susceptibility (Bull et al. 2014) in dependence of the susceptibility-switching rate and the cost of phenotypic resistance	Population dynamics for phase variation- (Kim and Ryu 2012; Turkington et al. 2019) and quorum sensing- (Høyland-Kroghsbo et al. 2013; Tan, Svenningsen, and Middelboe 2015) induced reduction in receptor number; percentage of non-adsorbed phages for receptor masking by phage T7 (Decker et al. 1994); plaque assays on cells with glycosylated (Harvey et al. 2018) or alanylated (Tzipilevich et al. 2022) receptors	Susceptibility-switching rates in the presence of phages; classification of differences in dynamics and subpopulation size for different mechanisms
Adsorption	Bacterial density-dependence of phage adsorption	Likely minor by itself as phage infection needs to be delayed until bacteria can escape by metabolic shutdown in the stationary phase but increases the likelihood of bacterial survival and coexistence in combination with phage density-dependence	Phage adsorption dependence on host growth rate at the population level through fitting to infection dynamics (Krysiak-Baltyn, Martin, and Gras 2018; Santos et al. 2014; Schrag and Mittler 1996); direct empirical measurements of growth rate dependence using different media (Golec et al. 2014; Hadas et al. 1997; Nabergoj, Modic, and Podgornik 2018)	Changes in adsorption heterogeneity within a population across growth phases; comparison between the effects of growth rate due to changes in media and due to changes in the growth phase
Adsorption	Saturation of infection efficiency at high phage densities	Increases the probability of coexistence in the absence of genetic resistance and in combination with bacterial density-dependence or fast phage decay also in the presence of genetic resistance	Population dynamics and molecular mechanisms of lysis inhibition, i.e. a prolonged time to lysis in coinfections of the same cell (Abedon 2019); Correlation between effective burst size and effective MOI (Gadagkar and Gopinathan 1980); Effect of MOI on burst size and latent period (Ellis and Delbrück 1939); Inference of infection saturation from bacterial killing in an *in vivo* mouse model (Roach et al. 2017)	The functional form of saturation of phage infection efficacy with MOI; intracellular effects of clonal phage coinfection other than lysis inhibition
Burst size	Bacterial density-dependence of the number of phage progeny produced	Likely minor under nutrient-limited conditions if phage infection is not delayed long enough for bacteria to enter the stationary phase	Burst size dependence on the host growth rate at the population level through fitting to infection dynamics (Choua and Bonachela 2019; Krysiak-Baltyn, Martin, and Gras 2018; Schrag and Mittler 1996); direct empirical measurements of growth rate dependence using different media (Birch, Ruggero, and Covert 2012; Golec et al. 2014; Hadas et al. 1997; Nabergoj, Modic, and Podgornik 2018; You, Suthers, and Yin 2002)	Changes in burst size heterogeneity within a population across growth phases
Latent period	Bacterial density-dependence of lysis timing	Likely minor (see burst size)	Latent period dependence on host growth rate at the population level through fitting to infection dynamics (Choua and Bonachela 2019; Krysiak-Baltyn, Martin, and Gras 2018); direct empirical measurements of growth rate dependence using different media (Birch, Ruggero, and Covert 2012; Golec et al. 2014; Hadas et al. 1997; Nabergoj, Modic, and Podgornik 2018; You, Suthers, and Yin 2002)	Changes in latent period heterogeneity within a population across growth phases
Latent period	Latent period distribution	Low variance of latent period distributions destabilizes coexistence	Latent period distributions through fitting to empirical data (Santos et al. 2014; Schrag and Mittler 1996); determination of stochasticity in lysis timing within a population in different growth conditions (Dennehy and Wang 2011)	Impact of phage density on latent period distribution and its correlation with burst size (other than lysis inhibition)
Decay	Intrinsic phage properties or environmental conditions	Faster decay increases the likelihood of bacterial survival and phage extinction	Intrinsic variation of average decay rates between different phages (Marianne and Taddei 2006); influence of environmental stressors on phage decay (Blazanin et al. 2022; Wommack et al. 1996)	Heterogeneity of decay within a phage population; Decay rate ranges in natural environments

Box 2. A general model to capture phenotypic flux in phage infection dynamics.

As empirical evidence shows that phenotypic variation can affect all phage infection processes, I propose a general model that can take into account various conceivable mechanisms. In this model, clonal bacteria and phage populations are subdivided according to their phenotypic infection traits into *i *= 1, …, *n* and *j *= 1, …, *m* classes, respectively. The metabolism of the *i*th bacterial class *B_i_* results in latent period *τ_i_* and burst size *β_i_*, whereas phage adsorption rate *α_i, j_* depends on bacterial (*i*) and phage (*j*) traits. *α_i, j_, β_i_*, and *τ_i_* can also depend on the total bacterial density *B* (e.g. bacterial growth phase) and the total phage density *P* (e.g. saturation of infection efficiency), which could be modeled as additional classes or through a functional dependence of these parameters on *B* and *P*. For simplicity, I assume that phages only adsorb to non-infected cells and avoid modeling infected classes explicitly (which could be included if adsorption leads to entering of another bacterial state). Note that it makes sense for latent period and burst size to change in a correlated manner between cell states, but that this is not necessarily true for adsorption rate.

Bacterial cells can switch from phenotypic class *i* to class *k* with rate *s_i, k_*. Each class *i* has a specific growth rate *r_i_*, growth cost *c_i_*, and death rate *γ_i_*. Phages are ‘born’ into a state *j*, which here determines the adsorption *α_i, j_* and the decay rate *ω_j_*, from the burst of any phage class *l *= 1, …, *m* at a proportion ${\epsilon_{l,j}}$ (with $\sum\limits_{j = 1, \ldots ,m} {{\epsilon_{l,j}}} = 1$).

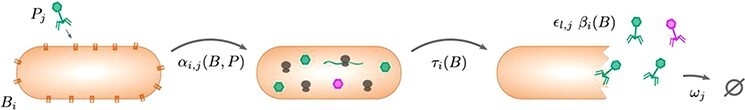

$$\frac{{d{B_i}}}{{dt}} = \underbrace {{r_i}\left( {1 - {c_i}} \right)\left( {1 - \sum\limits_{k = 1, \ldots ,n{\text{ }}k \ne i} {{s_{i,k}}} } \right){B_i}\left( {1 - \frac{B}{K}} \right)}_{ growth\;and\;switching\;to\;other\;physiological\;states}{\mkern 1mu} \; + \underbrace {\sum\limits_{k = 1, \ldots ,n{\text{ }}k \ne i} {{r_k}} \left( {1 - {c_k}} \right){s_{k,i}}\;{B_k}\left( {1 - \frac{B}{K}} \right)}_{switching\;from\;other\;physiological\;states}{\text{ }} - \underbrace {\sum\limits_{l = 1, \ldots ,m} {{\alpha _{i,l}}} \left( {B,P} \right){B_i}{P_l}}_{adsorption\;of\;{B_i}\;cells\;by\;all\;phage\;classes} - \underbrace {{\gamma _i}{B_i}}_{death}$$

$$\!\!\!\frac{{d{P_j}}}{{dt}} = \!\!\!\!\!\!\underbrace {\sum\limits_{l = 1, \ldots ,m} {\sum\limits_{{\kern 1pt} k = 1, \ldots ,n} {{\epsilon_{l,j}}} } {\beta _k}\left( B \right){\alpha _{k,l}}\left( {B,P} \right)\;{e^{ - {\gamma _k}{\tau _k}\left( B \right)}}\;{B_k}\left( {t - {\tau _k}\left( B \right)} \right)\;{P_l}\left( {t - {\tau _k}\left( B \right)} \right)}_{release\;of\;{P_j}\;phages\;from\;all\;bacterial\;classes\;that\;were\;infected\;with\;phage{\text{ }}classes\;that\;produce\;a\;proportion\;{\epsilon_{l,j}}\;of\;{P_j}\;phages} \!- \underbrace {\sum\limits_{k = 1, \ldots ,n} {{\alpha _{k,j}}} \left( {B,P} \right){B_k}{P_j}}_{adsorption\;to\;all\;bacterial\;classes} \!- \underbrace {\mathop {{\omega _j}{P_j}}\limits_{} }_{decay}$$

$$B = \sum\limits_{i = 1, \ldots ,n} {{B_i}} $$

$$P = \sum\limits_{j = 1, \ldots ,m} {{P_j}} $$

In addition, pathogen infections are complex environments, where the presence of other microbial species and the action of the immune system make phage–bacteria interactions and coexistence considerations even more complicated (Jover, Cortez, and Weitz 2013; Leung and Weitz 2017; Roach et al. 2017; Rodriguez-Gonzalez et al. 2020; Chevallereau et al. 2021). Notably, even the phages combined within a therapeutic cocktail might interfere with each other during infection of the same bacterial cell in unexpected ways (Schmerer, Molineux, and Bull 2014; Wright et al. 2021). Understanding the impact of phenotypic flux in low-diversity systems will also facilitate understanding in these more complex systems.

## Empirical quantification of phenotypic flux in phage infections

Phenotypic flux can be caused by any variation in phage or bacterial physiology, and we currently understand the mechanisms leading to phenotypic resistance only to a limited extent (Table 1). Especially for phenotypic resistance to phage adsorption, a range of mechanisms are available to bacteria, and different functional forms have been used, for example, to mathematically describe the dependence of adsorption rate on bacterial growth (Supplementary Equations (12–15); Schrag and Mittler 1996; Weitz and Dushoff 2008; Santos et al. 2014; Krysiak-Baltyn, Martin, and Gras 2018)). Hence, while incorporating phenotypic flux into mathematical models improves the fit with empirical data (Santos et al. 2014; Krysiak-Baltyn, Martin, and Gras 2018), the lack of mechanistic understanding limits the generality of these descriptions. The dependencies between phage and bacterial traits can be highly non-linear (Schwartz 1976), potentially not only shifting a trait value distribution but also changing its shape (Dennehy and Wang 2011). A general understanding would further require the mechanistic investigation of phenotypic flux across a diversity of phages and bacterial species, which might reveal novel ways in which phages deal with phenotypic flux in host populations.

As shown in Fig. 2, one crucial set of unknown parameters are the transition rates between bacterial cell states with different susceptibility to phage infection. These rates are likely dependent on the specific underlying mechanism and the phage trait affected, which could lead to a broad range of possible rates. Furthermore, bacterial states, and potential transitions between them, will change with growth rate and growth phase. Phage infection dynamics during phases of slow growth are however rarely studied, even though stationary phase or dormant cells likely constitute an important fraction of potential host cells encountered in nature (Chibani-Chennoufi et al. 2004). A systematic evaluation of bacterial cell states and their susceptibility to phages across different growth phases could provide crucial insights into phenotypic flux.

In trying to disentangle the contributions of different growth phases, but also more generally in studying phage–bacteria dynamics, the time frame of investigation and the sampling frequency are key. High sampling frequencies in the initial stages of the dynamics can help to accurately fit model parameters and to distinguish different mechanisms (Figs 2B, D vs. S2B, D). Furthermore, experimental measurements over several days might be necessary for the population dynamics to approximate a steady state and to detect biases introduced into mathematical models by certain assumptions (Lloyd 2001a).

Last but not least, finding a functional form for the variation in phage infection parameters is complicated by the fact that they often subsume several biological processes, which might not depend on physiological variation in the same manner. Instead, this complexity might call for a separation of model processes into several processes, which allows for more realism and mechanistic detail. By combining mathematical modeling and empirical data, it will then be possible to gauge the contribution of individual (sub-)processes to the overall infection dynamics and estimate the relevant parameters. Figure 2 suggests, for example, that the cost of phenotypic resistance determines the frequency and amplitude of oscillations in phage and bacterial populations, which could be used to estimate the costs indirectly from the overall dynamics. This approach is particularly useful as the costs and benefits of phenotypic traits are difficult to measure directly.

## Conclusion

In this review, I summarized the empirical evidence for substantial phenotypic variation in phage and bacterial traits within a population (Fig. 1). Mathematical descriptions of phenotypic flux in bacterial populations predict a decrease in the force of lytic phage infection that can lead to the coexistence of phages and susceptible bacteria as commonly found in empirical studies. The ‘ability’ of phages and their bacterial hosts to coexist under natural conditions could partly be an ‘in-built’ system property, resulting, for example, from intrinsic stochasticity and density-dependent effects that shape phage and bacterial physiology. Physiological interactions provide the most fundamental layer in phage–bacteria ecology and evolution. The whole picture then consists of multiple layers of interactions between phages and bacteria, ranging from physiological mechanisms to defense systems and genetic resistance to spatial effects and environmental transformation. It is therefore not surprising that predictions of phage infection dynamics over longer time scales remain challenging. Understanding first the individual layers and then connecting them will be crucial in aiding predictive efforts. These efforts are not only important to understand phage–bacteria ecology and evolution but also to make phages into an efficient tool against microbial infections. The results of phage therapy treatments are currently inconsistent and might be significantly improved by a better understanding of phage dependencies on bacterial cell physiology.

## Supplementary Material

veac086_Supp

## Data Availability

Equations and parameters used in the simulations are described in the main text or the supplementary material. Matlab Code (version R2019b) used for the simulations is available on request.
