# Supplementary material for: Phenotypic flux: The role of physiology in explaining the conundrum of bacterial persistence amid phage attack
Source: Virus Evol. 2022 Sep 15;8(2):veac086. doi: 10.1093/ve/veac086 (PMC9547521; doi:10.1093/ve/veac086)
Supplement: veac086_Supp [file veac086_supp.zip › Phage_review_SI.pdf]

Supplementary material for: Phenotypic flux: The role of physiology in explaining the conundrum of bacterial persistence amid phage attack

Claudia Igler[1*]

[1] Institute of Integrative Biology, Department of Environmental Systems Science, ETH Zürich, Zurich, Switzerland

[*] Corresponding author email: claudia.igler@env.ethz.ch

Supplementary Equations

Adsorption rate descriptions

- Phages can be heterogeneous in their ability to adsorb to bacterial cells (Storms and Sauvageau 2015), which can be incorporated into the model via a **low-adsorbing subpopulation of phages** P_l:

$$\frac{dS}{dt} = r\,(1-\mu)\,S\left(1-\frac{S+I+R}{K}\right) - \gamma S - \alpha SP \;-\; \alpha_l SP_l \tag{1}$$

$$\frac{dI}{dt} = \;\alpha SP \;+\; \alpha_l SP_l \;-\; \alpha e^{-\gamma\tau}S(t-\tau)P(t-\tau) \;-\; \alpha_l e^{-\,\gamma\tau}S(t-\tau)P_l(t-\tau) - \;\gamma I \tag{2}$$

$$\frac{dR}{dt} = r\,(1-c)\,R\left(1-\frac{S+I+R}{K}\right) + \mu r S\left(1-\frac{S+I+R}{K}\right) - \gamma R \tag{3}$$

$$\frac{dP}{dt} = \varepsilon\beta\alpha e^{-\,\gamma\tau}S(t-\tau)P(t-\tau) \;+ \varepsilon\beta\alpha_l e^{-\,\gamma\tau}S(t-\tau)P_l(t-\tau) \tag{4}$$

$$-\;\alpha(S+I)P \;-\; \omega P$$

$$\frac{dP_l}{dt} = (1-\varepsilon)\beta\alpha e^{-\,\gamma\tau}S(t-\tau)P(t-\tau) \;+\; (1-\varepsilon)\beta\alpha_l e^{-\,\gamma\tau}S(t-\tau)P_l(t-\tau) \tag{5}$$

$$-\;\alpha_l(S+I)P_l \;-\; \omega P_l$$

  This description assumes that the fraction of low-adsorbing phages stays the same at every burst, i.e. the same fraction ε of phages is adsorbing well, whereas a fraction $(1-\varepsilon)$ is adsorbing at rate $\alpha_l < \alpha$.

- Variation in **receptor density** can be modelled in two ways (Chapman-McQuiston and Wu 2008; Schenk and Sieber 2019):

  – Implicitly, by assuming that receptor density stays relatively constant at different growth rates and adsorption is mainly dependent on the total cell surface area (Schenk and Sieber 2019):

$$\alpha(r) = \frac{\alpha_{max} r^2}{d + r^2} \tag{6}$$

  where α_{max} is the maximal adsorption rate, r the bacterial growth rate and d the radius of the spherical bacterial cell.

- Explicitly, by making the adsorption rate directly dependent on the the number of receptors and introducing bacterial subpopulations $B_1, ..., B_n$ with receptor numbers $1, ..., n$ and adsorption rates $\alpha_1, ..., \alpha_n$ (Chapman-McQuiston and Wu 2008):

$$\alpha_n = \alpha_\infty \frac{ns}{ns + \frac{\pi a}{ln(2a/b)}} \tag{7}$$
$$\text{with } \alpha_\infty = \frac{4\pi Da}{ln(2a/b)}$$

where a and b are the major and minor semi-axis of the elliptical bacterial cell, D is the diffusion coefficient and s the radius of the receptor.

- Resistance via **phenotypic heterogeneity** describes transient bacterial subpopulations R that have lower adsorption rates caused by non-genetic mechanisms (Bull et al. 2014):

$$\begin{aligned}
\frac{dS}{dt} =& rS\left(1 - \frac{S+I+R}{K}\right) - \gamma S - \alpha SP - s_f rS\left(1 - \frac{S+I+R}{K}\right) \\
&+ s_b r\left(1-c\right)R\left(1 - \frac{S+I+R}{K}\right)
\end{aligned} \tag{8}$$

$$\frac{dI}{dt} = \alpha SP + \alpha_l RP - \alpha e^{-\gamma\tau}S(t-\tau)P(t-\tau) - \alpha_l e^{-\gamma\tau}R(t-\tau)P(t-\tau) - \gamma I \tag{9}$$

$$\begin{aligned}
\frac{dR}{dt} =& r\left(1-c\right)R\left(1 - \frac{S+I+R}{K}\right) - \gamma R - \alpha_l RP + s_f rS\left(1 - \frac{S+I+R}{K}\right) \\
&- s_b r\left(1-c\right)R\left(1 - \frac{S+I+R}{K}\right)
\end{aligned} \tag{10}$$

$$\begin{aligned}
\frac{dP}{dt} =& \beta\alpha e^{-\gamma\tau}S(t-\tau)P(t-\tau) + \beta\alpha_l e^{-\gamma\tau}R(t-\tau)P(t-\tau) - \omega P \\
&- \alpha(S+I)P - \alpha_l RP
\end{aligned} \tag{11}$$

The difference between a phenotypic R and a genetic R population is its stability. Switching between phenotypically resistant and susceptible states occurs at comparatively high rates, providing resources for phages to survive. Further, phenotypic resistance usually results in lower (but non-zero) adsorption rates α_l and, similar to genetic resistance, likely carries a cost.

Heterogeneity in adsorption on the bacterial side is different from that on the phage side (i.e. a low-adsorbing phage subpopulation) in that some bacterial cells are immediately protected from phage predation and provide a constant supply of susceptible cells, whereas the low-adsorbing phage fraction only affects dynamics when most phages have already adsorbed (i.e. most or all cells have already been killed).

- The dependence of phage infection success on **bacterial growth** is often incorporated into models by modifying the adsorption rate (Igler et al. 2022; Krysiak-Baltyn, Martin, and Gras 2018; Santos et al. 2014; Schrag and Mittler 1996; Weitz and Dushoff 2008):
  - With adsorption as a function of resource concentration (Schrag and Mittler 1996):

$$\alpha(C) = \alpha_S + f_\alpha\left(C\right)\left(\alpha_E - \alpha_S\right) \tag{12}$$

    where α_S and α_E are adsorption rates on stationary phase and exponential host cells respectively; and $f_\alpha\left(C\right)$ is a function between 0 and 1 that describes the dependence on resource concentration (e.g. as a function of the bacterial growth rate).

- Using a logistic function of bacterial density (Igler et al. 2022; Weitz and Dushoff 2008; for a generalization of this approach see Wang and Goldenfeld 2010):

$$\alpha S\left(1-f\frac{S}{K}\right)^{k_2}P \tag{13}$$

where f is the fractional reduction in lysis at carrying capacity K and k_2 is the power-law exponent of the density dependence (k_2=1 in Weitz and Dushoff 2008).

- Using an exponential function of growth rate with explicit resource consideration (Santos et al. 2014):

$$\alpha(C)=\alpha_{min}e^{\lambda r(C)} \tag{14}$$

where r is the bacterial growth rate, C the resource concentration and α_{min} and λ empirically fitted scaling parameters describing the minimal adsorption rate and the exponential factor of the growth rate-dependence of adsorption.

- Using an exponential function of growth rate without explicit resource consideration (Krysiak-Baltyn, Martin, and Gras 2018):

$$\alpha(r)=\alpha_{min}10^{\lambda\frac{r}{r_{max}}} \tag{15}$$

where α_{min} is the minimal adsorption rate, r_{max} the maximal growth rate and λ the exponential factor of the growth rate-dependence of adsorption.

• **Saturation of adsorption** at high phage densities (i.e. reduction in adsorption gains with more phages infecting one cell) (Rodriguez-Gonzalez et al. 2020; Smith 2008; Stopar and Abedon 2009) can be described:

- Using an exponential function of phage density (Stopar and Abedon 2009):

$$\left(1-e^{-\alpha P}\right)S \tag{16}$$

- Using a saturating Hill function of phage density (Rodriguez-Gonzalez et al. 2020):

$$\alpha\frac{1}{1+\frac{P}{P_c}}SP \tag{17}$$

where P_c is the phage density at which the infection rate is half saturated. For a similar approach, where P_c is given by the inverse ratio of phage adsorption and DNA injection time, see Smith 2008.

• **Heterogeneous mixing** of phages and bacteria (Rodriguez-Gonzalez et al. 2020) can be approximated by:

$$\tilde{\alpha}SP^{\gamma} \tag{18}$$

where $\tilde{\alpha}$ is the nonlinear adsorption rate and $\gamma<1$ is the power-law exponent.

Biosynthesis parameter descriptions

Burst size

- Burst size can be described as a function of **bacterial growth** in different ways (Choua and Bonachela 2019; Krysiak-Baltyn, Martin, and Gras 2018; Nabergoj, Modic, and Podgornik 2018; Schenk and Sieber 2019; Schrag and Mittler 1996):
  - With burst size as a linear function of growth rate (Nabergoj, Modic, and Podgornik 2018; Schenk and Sieber 2019):

$$\beta(r) = dr \tag{19}$$

    where d is the scaling constant for the growth rate-dependence. (The impact of the growth rate r can also be described relative to the maximal growth rate r_{max} (Krysiak-Baltyn, Martin, and Gras 2018).)
  - With burst size as a function of growth rate through the sigmoidal dependence of maturation rate on growth rate (Choua and Bonachela 2019):

$$\beta(r) = \frac{m(r)}{\gamma} \tag{20}$$

    where $m(r)$ is the growth-dependent maturation rate of virions inside the cell and γ the removal rate of infected hosts (or more generally, removal of intracellular virions).
  - With burst size as a function of resource concentration (Schrag and Mittler 1996):

$$\beta(C) = \beta_S + f_\beta(C)(\beta_E - \beta_S) \tag{21}$$

    where β_S and β_E are burst sizes on stationary phase and exponential host cells respectively; and $f_\beta(C)$ is a function between 0 and 1 that describes the dependence on resource concentration (e.g. as a function of the bacterial growth rate).

Latent period

- Latent period can also be described as a function of **bacterial growth** (Choua and Bonachela 2019; Krysiak-Baltyn, Martin, and Gras 2018; Nabergoj, Modic, and Podgornik 2018):
  - With latent period as a linear function of growth rate (Krysiak-Baltyn, Martin, and Gras 2018):

$$\tau(r) = \tau_{max} - \tau_{min}\frac{r}{r_{max}} \tag{22}$$

    where τ_{max} and τ_{min} are the maximal and minimal latent period respectively; and r_{max} the maximal growth rate.
  - Using a saturating Hill function (Nabergoj, Modic, and Podgornik 2018):

$$\tau(r) = \frac{K_{lat} + r}{\frac{1}{L_{min}} r} \tag{23}$$

    where L_{min} is the minimal latent period and K_{lat} the latent period at half-saturation.
  - With latent period as a function of growth rate through the decreasing exponential dependence of the eclipse period (the time from infection to the first assembled virion inside the cell) on growth rate (Choua and Bonachela 2019):

$$\tau(r) = \frac{1}{\gamma} + E(r) \tag{24}$$

    where $E(r)$ is the growth-dependent eclipse period and γ the removal rate of infected hosts (or more generally, removal of intra-cellular virions).

- The variation in lysis timing of individual cells can be described by using **latent period distributions** ((Lloyd 2001; Santos et al. 2014; Schrag and Mittler 1996):
  - Using the method of stages to obtain a realistic distribution of latent periods (Lloyd 2001):

$$\frac{dS}{dt} = r\,(1-\mu)\,S\left(1 - \frac{S + I_1 + ... + I_n + R}{K}\right) - \gamma S - \alpha SP \tag{25}$$

$$\frac{dI_1}{dt} = \alpha SP - nt_r I_1 \tag{26}$$

$$\frac{dI_2}{dt} = nt_r I_1 - nt_r I_2 \tag{27}$$

$$...$$

$$\frac{dI_n}{dt} = nt_r I_{n-1} - nt_r I_n \tag{28}$$

$$\frac{dR}{dt} = r\,(1-c)\,R\left(1 - \frac{S + I_1 + ... + I_n + R}{K}\right) \tag{29}$$

$$+ \mu r S\left(1 - \frac{S + I_1 + ... + I_n + R}{K}\right) - \gamma R \tag{30}$$

$$\frac{dP}{dt} = \beta nt_r I_n - \ \alpha(S + I_1 + ... + I_n)P \ - \ \omega P \tag{31}$$

  where $I_1, ..., I_n$ describe the n infected stages and t_r the lysis rate. The total time spent within the cell (from I_1 to I_n), i.e. the latent period, is given by a sum of n exponential distributions, leading to a gamma distribution (which becomes closer to a normal distribution with increasing number of stages n). Hence, by using nt_r as the transition rate between infected stages, the mean latent period is kept the same ($\frac{1}{t_r}$), but the variation decreases with the number of stages ($\frac{1}{nt_r^2}$). I assume here that only the last infected stage can produce a phage burst of β virions, which is the only way by which infected cells die. This can be a reasonable assumption for many lytic phages under optimal conditions, where latent periods can be as short as 13min (De Paepe and Taddei 2006), but this assumption does not affect the outcome strongly. Note that the infected stages do not necessarily correspond to biological processes in phage infection. A description and analysis of a more general 'infection-age' model with distributed latent periods and burst sizes can be found in Smith and Thieme 2012.

  - Describing asynchronous bursting using a uniform distribution of lysis events (Schrag and Mittler 1996):

$$\frac{\beta}{\tau_{min} - \tau_{max}} \alpha \sum_{\tau=\tau_{min}}^{\tau_{max}} S(t-\tau)P(t-\tau)e^{-\gamma\tau}d\tau \tag{32}$$

  where the bursts are uniformly distributed between the minimal latent period (first burst) τ_{min} and the maximal latent period (last burst) τ_{max}, and summed up over the whole time period in between. The probability of a cell bursting after τ time units gives the weight for the number of released phages in this summation and could be adjusted to represent any other distribution by replacing $\frac{1}{\tau_{min} - \tau_{max}}$ with another probability density function. If the adsorption rate is found to be time-dependent as well, it can be included inside the sum as $\alpha(t-\tau)$.

  - Describing a softer rise of phages at the beginning of the epidemic using a normal distribution (Santos et al. 2014):

$$B(\tau) = \alpha S(t-\tau)P(t-\tau)N(\tau) \tag{33}$$

  gives the number of bacteria that have a latent period of τ, where $N(\tau)$ describes a normal distribution governing the latent period $\tau \in \{\tau_{min}, \tau_{max}\}$. The number of bacteria that will

burst at time t is then given by the sum over the bacteria with latent period τ:

$$\beta \sum_{\tau=\tau_{min}}^{\tau_{max}} B(\tau) \tag{34}$$

See Rabinovitch et al. 2002 for another use of a normal distribution to describe lysis timing variation.

Supplementary Figures and Tables

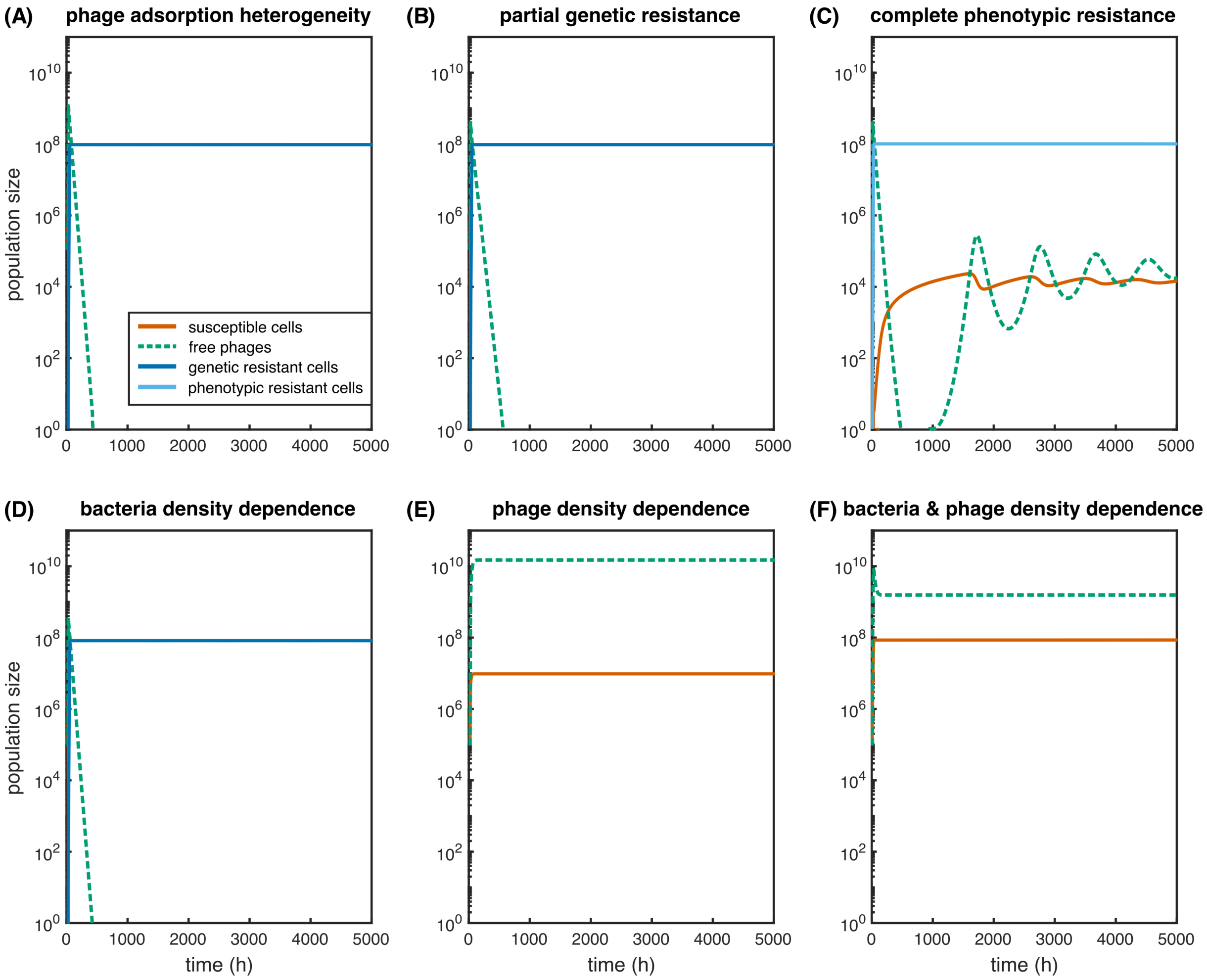

Figure S1: **Phage-bacteria population dynamics considering additional types of phenotypic flux in phage adsorption.** Phage and bacterial numbers (PFU/ml and CFU/ml) were simulated for 5000h starting from an initial MOI=1 (other parameters are given in Table S1). Population dynamics are shown for (A) phages with low- and high-adsorbing subpopulations; (B) genetic resistance that only partially protects from phages; (C) phenotypic resistance with complete protection and without cost; (D) bacterial density-dependent adsorption; (E) phage-density-dependent adsorption without resistance evolution; and (F) phage- and bacterial density-dependent adsorption without resistance evolution. Orange indicates susceptible cells, dashed green free phages, dark blue genetically resistant cells in A,E,F; and light blue phenotypically resistant cells in B-D.

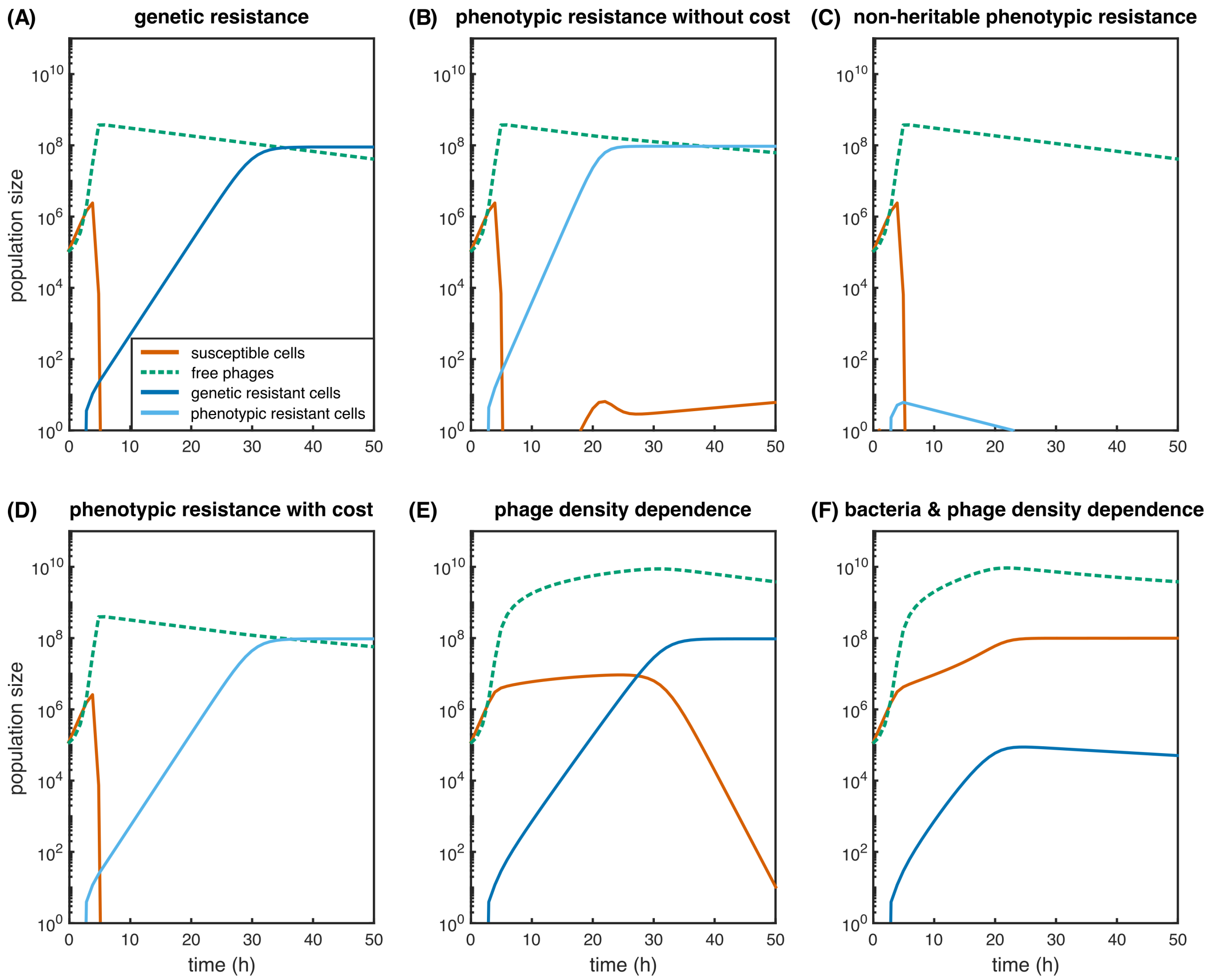

Figure S2: **Phage-bacteria population dynamics considering phenotypic flux in phage adsorption.** Phage and bacterial numbers (PFU/ml and CFU/ml) were simulated for 50h starting from an initial multiplicity of infection (MOI)=1 (other parameters are given in Table S1). Population dynamics are shown for constant adsorption rate with (A) genetic (stable) resistance; (B,C) phenotypic resistance without cost and switching of bacterial cells between high- and low-adsorbable states at (B) intermediate or (C) fast rates; and (D) phenotypic resistance with intermediate switching and growth cost. (E) and (F) show genetic resistance evolution when adsorption rate is (E) phage- or (F) phage- and bacterial density-dependent. Orange indicates susceptible cells, dashed green free phages, dark blue genetically resistant cells in A,E,F; and light blue phenotypically resistant cells in B-D.

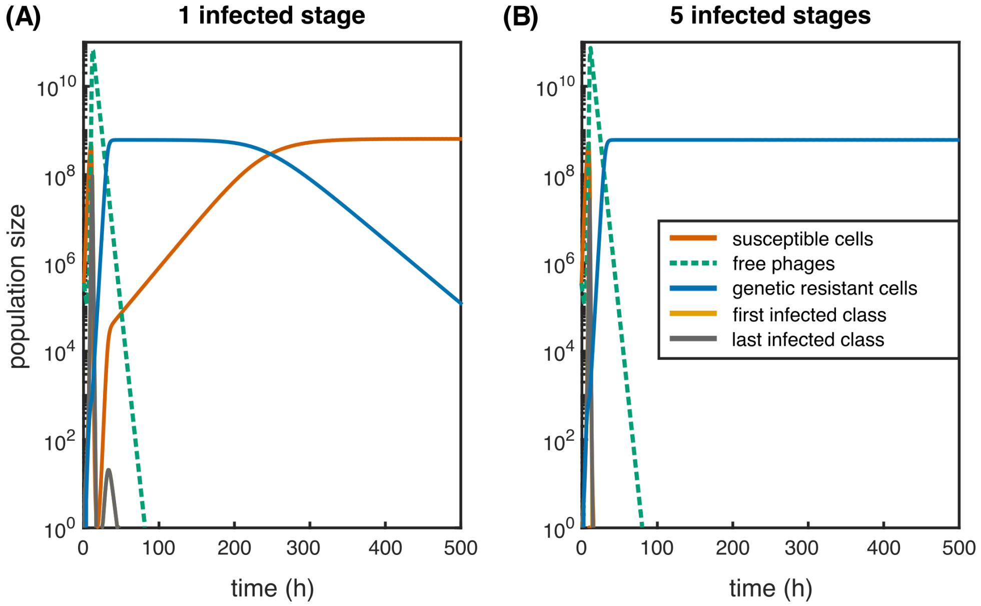

Figure S3: **Phage-bacteria population dynamics for realistic latent period distributions with resistance evolution.** Phage and bacterial numbers (PFU/ml and CFU/ml) were simulated for 500h starting from an initial MOI=1 (parameters are given in Table S1). Population dynamics are shown for gamma-distributed latent periods with (A) 1 or (B) 5 infected stages with resistance evolution. For these simulations lower adsorption rate ($\alpha = 10^{-9}h^{-1}$) and higher phage decay rate ($\omega = 0.33h^{-1}$) were used. Orange indicates susceptible cells, dashed green free phages and dark blue genetically resistant cells. Yellow shows the first infected stage and grey the last one.

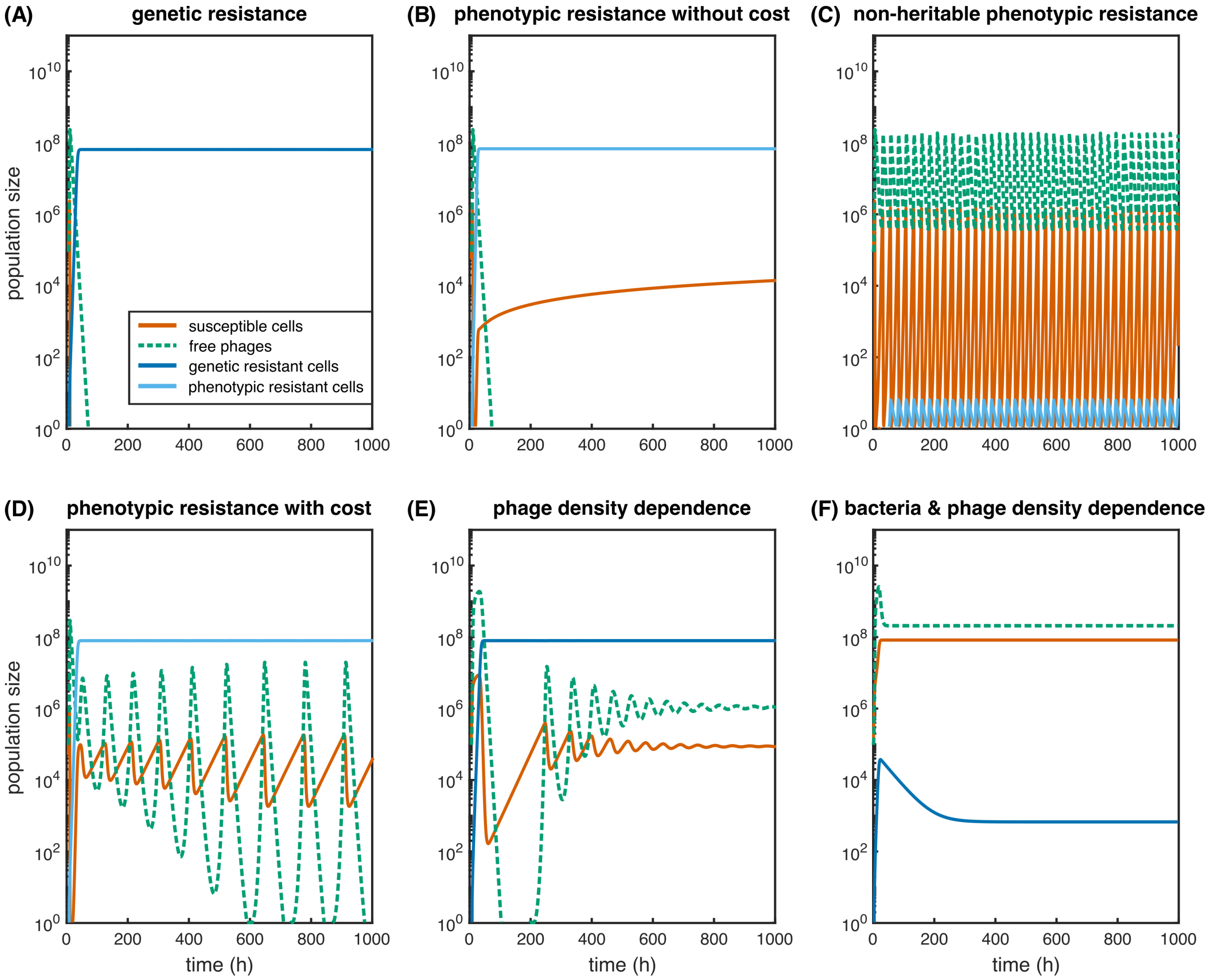

Figure S4: **Phage-bacteria population dynamics considering phenotypic flux in phage adsorption for faster phage decay.** Phage and bacterial numbers (PFU/ml and CFU/ml) were simulated for 1000h starting from an initial multiplicity of infection (MOI)=1 using a faster phage decay rate $\omega = 0.33h^{-1}$ (other parameters are given in Table S1). Population dynamics are shown for constant adsorption rate with (A) genetic (stable) resistance; (B,C) phenotypic resistance without cost and switching of bacterial cells between high- and low-adsorbable states at (B) intermediate or (C) fast rates; and (D) phenotypic resistance with intermediate switching and growth cost. (E) and (F) show genetic resistance evolution when adsorption rate is (E) phage- or (F) phage- and bacterial density-dependent. Orange indicates susceptible cells, dashed green free phages, dark blue genetically resistant cells in A,E,F; and light blue phenotypically resistant cells in B-D.

Table S1: Variables and parameters used in simulations of the mathematical model shown in Box 1. Values in brackets indicate parameters used for the model version implementing the 'method of stages'.

Variables and parameters	Values	Description	Source
S	10^5 cells	Initial number of susceptible bacteria	This paper
R	0 cells	Initial number of genetic or phenotypically phage-resistant bacteria	This paper
P	10^5 virions	Initial number of phage virions	This paper
r	1h^{-1}	Growth rate of susceptible cells	This paper
c	0.3	Growth cost of genetic or phenotypically phage-resistant cells	This paper
K	10^8 cells	Carrying capacity	This paper
γ	0.1h^{-1}	Bacterial death rate	Berngruber, Weissing, and Gandon 2010
ω	0.05h^{-1} (0.33h^{-1})	Virion decay rate	adapted from De Paepe and Taddei 2006
α	$4 \cdot 10^{-8}$mL h^{-1} (10^{-9}mL h^{-1})	Adsorption rate of phages to bacterial cells	Pleska et al. 2018
α_l	10^{-10}mL h^{-1}	Adsorption rate of phages with low adsorption efficacy or to phenotypically resistant bacterial cells	Bull et al. 2014
β	100 virions	Burst size (number of phage virions released)	Berngruber, Weissing, and Gandon 2010
k_2	2	Power-law exponent for growth rate dependence of phage adsorption and burst size	Igler et al. 2022
τ	0.3h	Latent period	Cortes et al. 2017
μ, s_f, s_b	$1.7 \cdot 10^{-6}$h^{-1}	Mutation and switchting rate for genetic or phenotypic resistance	Bull et al. 2014
ε	0.4	Fraction of non-absorbing phages	Storms and Sauvageau 2015
P_c	$0.2 \cdot 10^8$	Phage concentration at half-saturation of adsorption	Rodriguez-Gonzalez et al. 2020

References

Berngruber, Thomas W, Franz J Weissing, and Sylvain Gandon (2010). “Inhibition of Superinfection and the Evolution of Viral Latency”. In: *Journal of Virology* 84.19, pp. 10200–10208. ISSN: 0022-538X. DOI: 10.1128/JVI.00865-10. URL: http://jvi.asm.org/cgi/doi/10.1128/JVI.00865-10.

Bull, James J. et al. (2014). “Phenotypic Resistance and the Dynamics of Bacterial Escape from Phage Control”. In: *PLoS ONE* 9.4. Ed. by Daniel E. Rozen, e94690. ISSN: 1932-6203. DOI: 10.1371/journal.pone.0094690.

Chapman-McQuiston, E. and X. L. Wu (2008). “Stochastic receptor expression allows sensitive bacteria to evade phage attack. Part II: Theoretical analyses”. In: *Biophysical Journal* 94.11, pp. 4537–4548. ISSN: 15420086. DOI: 10.1529/biophysj.107.121723.

Choua, Melinda and Juan A. Bonachela (2019). “Ecological and evolutionary consequences of viral plasticity”. In: *American Naturalist* 193.3, pp. 346–358. ISSN: 00030147. DOI: 10.1086/701668.

Cortes, M. G. et al. (2017). “Late-Arriving Signals Contribute Less to Cell-Fate Decisions”. In: *Biophys J* 113.9, pp. 2110–2120. ISSN: 1542-0086 (Electronic) 0006-3495 (Linking). DOI: 10.1016/j.bpj.2017.09.012. URL: https://www.ncbi.nlm.nih.gov/pubmed/29117533.

De Paepe, Marianne and François Taddei (2006). “Viruses’ life history: Towards a mechanistic basis of a trade-off between survival and reproduction among phages”. In: *PLoS Biology* 4.7, pp. 1248–1256. ISSN: 15457885. DOI: 10.1371/journal.pbio.0040193.

Igler, Claudia et al. (2022). “Conjugative plasmid transfer is limited by prophages but can be overcome by high conjugation rates”. In: *Philosophical Transactions of the Royal Society B: Biological Sciences* 377.1842, p. 2021.03.29.437513. ISSN: 0962-8436. DOI: 10.1098/rstb.2020.0470. URL: https://doi.org/10.1101/2021.03.29.437513%20https://royalsocietypublishing.org/doi/10.1098/rstb.2020.0470.

Krysiak-Baltyn, Konrad, Gregory J.O. Martin, and Sally L. Gras (2018). “Computational modelling of large scale phage production using a two-stage batch process”. In: *Pharmaceuticals* 11.2, pp. 1–14. ISSN: 14248247. DOI: 10.3390/ph11020031.

Lloyd, Alun L. (2001). “Destabilization of epidemic models with the inclusion of realistic distributions of infectious periods”. In: *Proceedings of the Royal Society B: Biological Sciences* 268.1470, pp. 985–993. ISSN: 14712970. DOI: 10.1098/rspb.2001.1599.

Nabergoj, Dominik, Petra Modic, and Aleš Podgornik (2018). “Effect of bacterial growth rate on bacteriophage population growth rate”. In: *MicrobiologyOpen* 7.2, e00558. ISSN: 20458827. DOI: 10.1002/mbo3.558.

Pleska, M. et al. (2018). “Phage-host population dynamics promotes prophage acquisition in bacteria with innate immunity”. In: *Nat Ecol Evol* 2.2, pp. 359–366. ISSN: 2397-334X (Electronic) 2397-334X (Linking). DOI: 10.1038/s41559-017-0424-z. URL: https://www.ncbi.nlm.nih.gov/pubmed/29311700.

Rabinovitch, Avinoam et al. (2002). “Bacteriophage T4 development in Escherichia coli is growth rate dependent”. In: *Journal of Theoretical Biology* 216.1, pp. 1–4. ISSN: 00225193. DOI: 10.1006/jtbi.2002.2543.

Rodriguez-Gonzalez, Rogelio A. et al. (2020). “Quantitative Models of Phage-Antibiotic Combination Therapy”. In: *mSystems* 5.1. Ed. by Katrine L. Whiteson. ISSN: 2379-5077. DOI: 10.1128/mSystems.00756-19. URL: http://msystems.asm.org/lookup/doi/10.1128/mSystems.00756-19.

Santos, Sílvio B. et al. (2014). “Population Dynamics of a Salmonella Lytic Phage and Its Host: Implications of the Host Bacterial Growth Rate in Modelling”. In: *PLoS ONE* 9.7. Ed. by Julio Vera, e102507. ISSN: 1932-6203. DOI: 10.1371/journal.pone.0102507.

Schenk, Hanna and Michael Sieber (2019). “Bacteriophage can promote the emergence of physiologically sub-optimal host phenotypes”. In: pp. 1–13. DOI: 10.1101/621524.

Schrag, S.J and J. E. Mittler (1996). “Host-Parasite Coexistence : The Role of Spatial Refuges in Stabilizing Bacteria-Phage Interactions Author (s): S . J . Schrag and J . E . Mittler Source : The American Naturalist , Aug ., 1996 , Vol . 148 , No . 2 (Aug ., 1996), pp . 348-377 Published”. In: *The American naturalist* 148.2, pp. 348–377.

Smith, Hal L. (2008). “Models of Virulent Phage Growth with Application to Phage Therapy”. In: *SIAM Journal on Applied Mathematics* 68.6, pp. 1717–1737. ISSN: 0036-1399. DOI: 10.1137/070704514. URL: http://epubs.siam.org/doi/10.1137/070704514.

Smith, Hal L. and Horst R. Thieme (2012). “Persistence of bacteria and phages in a chemostat”. In: *Journal of Mathematical Biology* 64.6, pp. 951–979. ISSN: 03036812. DOI: 10.1007/s00285-011-0434-4.

Stopar, David and Stephen T. Abedon (2009). “Modeling bacteriophage population growth”. In: *Bacteriophage Ecology*, pp. 389–414. DOI: 10.1017/cbo9780511541483.018.

Storms, Zachary J. and Dominic Sauvageau (2015). “Modeling tailed bacteriophage adsorption: Insight into mechanisms”. In: *Virology* 485, pp. 355–362. ISSN: 00426822. DOI: 10.1016/j.virol.2015.08.007.

Wang, Zhenyu and Nigel Goldenfeld (2010). “Fixed points and limit cycles in the population dynamics of lysogenic viruses and their hosts”. In: *Physical Review E - Statistical, Nonlinear, and Soft Matter Physics* 82.1, pp. 1–18. ISSN: 15393755. DOI: 10.1103/PhysRevE.82.011918. arXiv: 1003.2658.

Weitz, Joshua S and Jonathan Dushoff (2008). “Alternative stable states in host–phage dynamics”. In: *Theoretical Ecology* 1.1, pp. 13–19. ISSN: 1874-1738. DOI: 10.1007/s12080-007-0001-1. URL: http://link.springer.com/10.1007/s12080-007-0001-1.
